# Metabolic tuning of a stable microbial community in the surface oligotrophic Indian Ocean revealed by integrated meta-omics

**DOI:** 10.1007/s42995-021-00119-6

**Published:** 2022-01-01

**Authors:** Zhang-Xian Xie, Ke-Qiang Yan, Ling-Fen Kong, Ying-Bao Gai, Tao Jin, Yan-Bin He, Ya-Yu Wang, Feng Chen, Lin Lin, Zhi-Long Lin, Hong-Kai Xu, Zong-Ze Shao, Si-Qi Liu, Da-Zhi Wang

**Affiliations:** 1 State Key Laboratory of Marine Environmental Science/College of the Environment and Ecology, Xiamen University, Xiamen, 361005 China; 2 College of Ocean and Earth Sciences, Xiamen University, Xiamen, 361005 China; 3 Southern Marine Science and Engineering Guangdong Laboratory (Zhuhai), Sun Yat-Sen University, Zhuhai, 519082 China; 4 BGI-Shenzhen, Beishan Industrial Zone 11th Building, Shenzhen, 518083 China; 5 BGI Education Center, University of Chinese Academy of Sciences, Shenzhen, 518083 China; 6 Key Laboratory of Marine Genetic Resources, Third Institute of Oceanography, Ministry of Natural Resources of China, Xiamen, 361005 China; 7 State Key Laboratory Breeding Base of Marine Genetic Resources/Fujian Key Laboratory of Marine Genetic Resources, Xiamen, 361005 China; 8 Institute of Marine and Environmental Technology, University of Maryland Center for Environmental Science, Baltimore, MD 21202 USA

**Keywords:** Microbial community, Metabolic tuning, Metaproteomics, Metagenomics, Oligotrophic ocean

## Abstract

Understanding the mechanisms, structuring microbial communities in oligotrophic ocean surface waters remains a major ecological endeavor. Functional redundancy and metabolic tuning are two mechanisms that have been proposed to shape microbial response to environmental forcing. However, little is known about their roles in the oligotrophic surface ocean due to less integrative characterization of community taxonomy and function. Here, we applied an integrated meta-omics-based approach, from genes to proteins, to investigate the microbial community of the oligotrophic northern Indian Ocean. Insignificant spatial variabilities of both genomic and proteomic compositions indicated a stable microbial community that was dominated by *Prochlorococcus*, *Synechococcus*, and SAR11. However, fine tuning of some metabolic functions that are mainly driven by salinity and temperature was observed. Intriguingly, a tuning divergence occurred between metabolic potential and activity in response to different environmental perturbations. Our results indicate that metabolic tuning is an important mechanism for sustaining the stability of microbial communities in oligotrophic oceans. In addition, integrated meta-omics provides a powerful tool to comprehensively understand microbial behavior and function in the ocean.

**Supplementary Information:**

The online version contains supplementary material available at 10.1007/s42995-021-00119-6.

## Introduction

Oligotrophic oceans, which are characterized by extremely low nutrient and chlorophyll concentrations, cover 60% of the ocean surface and contribute more than 30% of global marine primary production, thereby playing a central role in regulating biogeochemical cycling and the global climate (Marañón et al. 2003). Recent global cultivation-independent molecular surveys have revealed that microbes are diverse and abundant in oligotrophic oceans, they drive biogeochemical processes, such as carbon and nutrients cycling, and shape the entire ecosystem (Azam and Malfatti 2007; Rusch et al. 2007; Sunagawa et al. 2015).

Over the past few decades, growing evidence has accumulated that the composition and diversity of microbial communities vary with environmental change (Dupont et al. 2014; Fortunato and Crump 2015; Herlemann et al. 2011; Ortega-Retuerta et al. 2013; Yoshimura et al. 2018), and that microbial community structure and function at the surface are mainly driven by salinity and temperature (Sunagawa et al. 2015; Zheng et al. 2016). However, interestingly, some studies have shown that the taxonomic composition of these communities does not directly lead to metabolic variation in all marine environments (Fortunato and Crump 2015; Sunagawa et al. 2015), suggesting a decoupling between taxonomy and function in response to environmental perturbations (Louca et al. 2018). It remains unclear how much changes in community structure would affect microbial function in oligotrophic oceans and what roles such a decoupling would play in shaping the structure and function of the community.

Studies of microbial ecosystems have shown that the link between community composition and metabolic response is strongly influenced by functional redundancy and metabolic tuning (Louca et al. 2018; Moya and Ferrer 2016). Functional redundancy is the coexistence of distinct species that have the same metabolic function, while metabolic tuning describes a community that is able to acclimate to environmental changes by adjusting overall metabolism. These two properties influence how microbial communities respond to environmental changes and modulate the covariation between community composition and functional outcome (Moya and Ferrer 2016). However, little is known about how these properties mediate microbial composition and function in oligotrophic oceans. While current marine meta-omics studies, such as metagenomics, metatranscriptomics and metaproteomics, provide valuable information on both the taxonomic and functional composition of natural microbial communities (Bergauer et al. 2018; De Long et al. 2006; Frias-Lopez et al. 2008; Morris et al. 2010; Rusch et al. 2007; Sowell et al. 2011; Sunagawa et al. 2015; Wang et al. 2013), almost all these studies characterize microbes solely on one molecular level (DNA, message RNA or protein), which impedes a comprehensive understanding of structuring mechanisms and their stability in the ocean. Therefore, there is a need to go beyond taxonomy and to devote more effort to integrating taxonomy, functional potential and metabolic activity to reveal the mechanisms underlying environmental structuring of microbial community in oligotrophic oceans.

The northern Indian Ocean, with a significant east–west salinity gradient, is a highly productive region (Du and Zhang 2015). However, the structuring of microbial community by environmental factors, such as salinity, in this ecologically important biogeochemical province remains to be settled. The present study investigated the microbial metabolic potential and activity across the northern Indian Ocean using an integrated meta-omic (metagenomic and metaproteomic) approach, aiming to facilitate a holistic understanding on how microbial metabolic function co-varies with environmental perturbation to maintain ecosystem stability in the surface ocean. Our results indicate that metabolic tuning occurring at different molecular levels sustains the stability of the microbial community in the northern Indian Ocean.

## Results

### Overview of metagenomics and metaproteomics

**Fig. 1:**
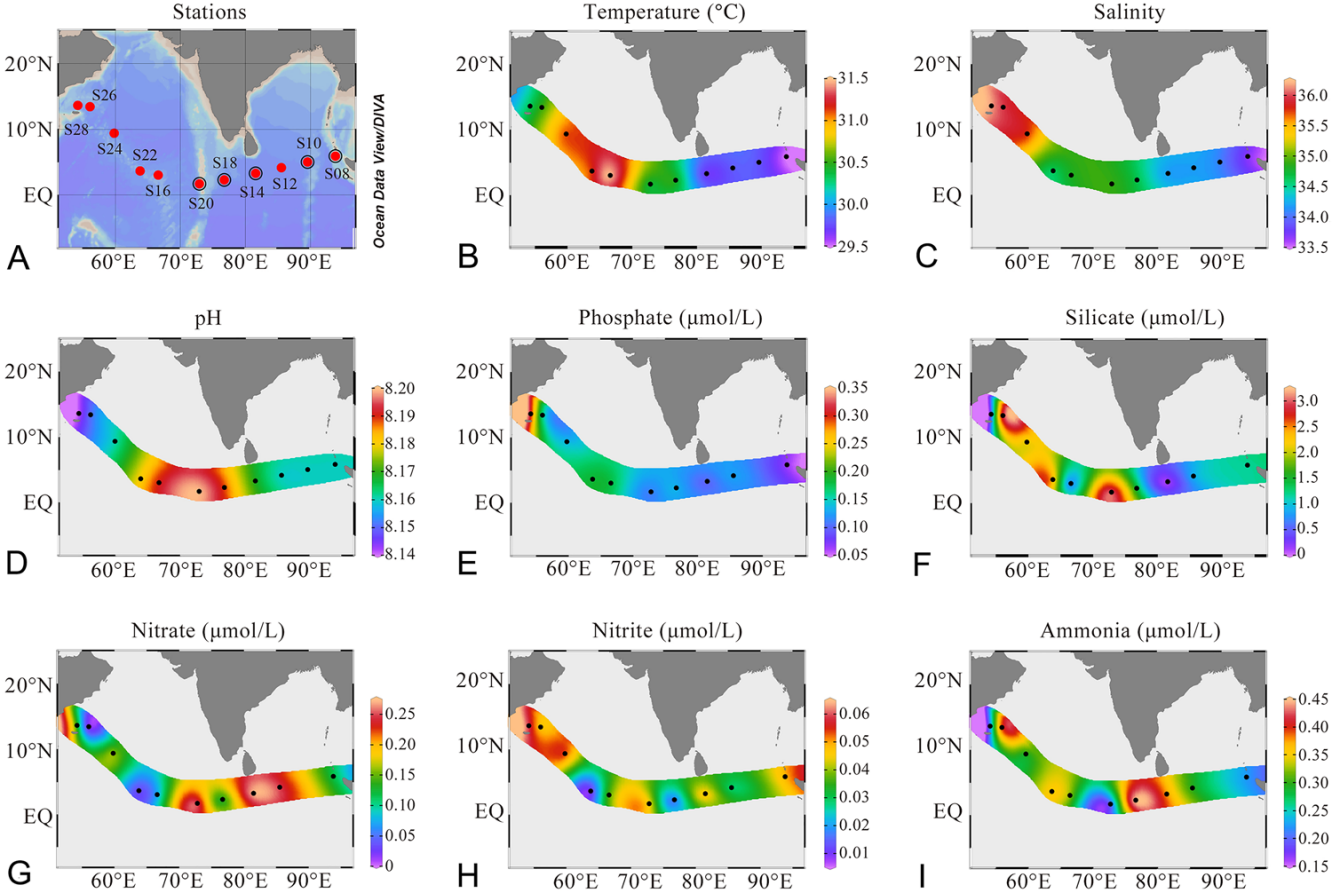
Spatial distribution of environmental factors and sampling stations in the Indian Ocean (figures are created based on data in Supplementary Table S1 with the software of Ocean Data View)

Both the genomic and proteomic composition of the same microbial community (within the size fraction between 0.2 and 1.6 µm) from 11 sites across a surface transect of the northern Indian Ocean were examined (Fig. 1A and Supplementary Table S1). Single-end 50 bp (SE50) sequencing was applied to each sample with an output of 719–850 million clean reads (Supplementary Table S2). This strategy was followed because the global Ocean Microbial Reference Gene Catalog (OM-RGC) includes most of the genetic diversity in the global ocean microbiome (Sunagawa et al. 2015) and single-end reads with a length of 50 bp is long enough for the detection of different genes (Chhangawala et al. 2015). On average, 73.2 ± 2.8% of these reads could be mapped to the OM-RGC dataset (Sunagawa et al. 2015). A range of 2.3–3.9 million genes from the OM-RGC were recovered and quantitative analysis was conducted. The peptide mixtures were subjected to the LC–MS/MS-based shotgun metaproteomic analysis. Each sample generated 291,713 ± 32,976 spectra and was interpreted using a three-step search (see materials and methods) against a predicted protein dataset of SE50-recovered OM-RGC genes (Supplementary Table S3). An average of 27,821 ± 2442 peptides and 5622 ± 331 proteins were confidently identified per sample and more than 90% of the peptides from each sample had high qualities for quantification.

### Microbial community composition

**Fig. 2:**
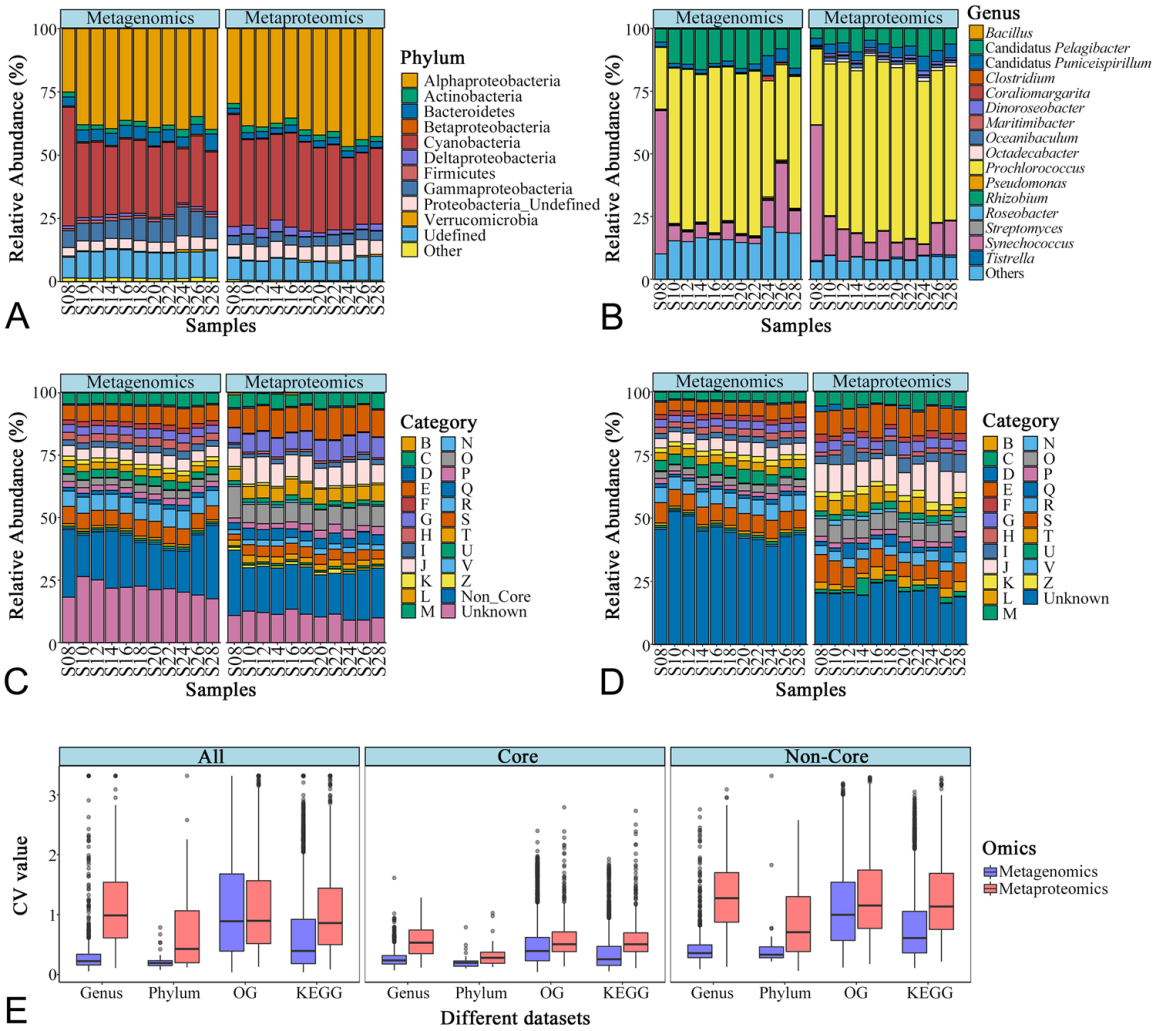
Gene- and protein-centric structure and spatial variability of the microbial community in the oligotrophic Indian Ocean. **A** and **B** show microbial compositions at the phylum (class for *Proteobacteria*, (**A)**) and genus (**B**) levels based on the taxonomic assignment of metagenome-predicted functional genes or metaproteome-detected proteins; **C** and **D** show functional distribution in terms of COG categories using all proteins that have OG assignments (**C**) and their non-core subsets, which comprise of OGs not present in all sites (**D**, grey colored in **C**); coefficient variations in **E** describe spatial variability of taxonomic and functional compositions in the community using different subsets of omics data, such as all genes/proteins, and core and non-core OGs

The total and active microbial communities were assessed by taxonomic binning of the protein homologs present in the metagenomic and metaproteomic datasets (Fig. 2A, B). More than 85% and 95% of gene and protein abundance in the metagenome and metaproteome, respectively, had taxonomic annotations (Supplementary Tables S2, S3). Of the taxonomic categories, bacteria contributed the most to the genomic and proteomic composition (74.9 ± 3.6% and 90.8 ± 1.5%, respectively; Supplementary Tables S2, S3). The pairwise Spearman’s correlation analysis of gene and protein abundances indicated a similar bacterial composition at the phylum level between total (metagenomics) and active (metaproteomic) communities (*R* = 0.923, *P* < 0.05). Furthermore, a similar bacterial composition, identified either from the total or active community, was observed at the different sampling sites (Fig. 2A). *Alphaproteobacteria* and *Cyanobacteria* were the dominant and active bacteria at each site. When examined at the genus level, such similarity was also observed in most groups, except for *Synechococcus*, which was abundant at site S8 (Fig. 2B). *Prochlorococcus*, *Synechococcus*, *Candidatus* Pelagibacter (SAR11) and *Candidatus* Puniceispirillum (SAR116) were the most abundant genera in both the total and active communities (Fig. 2B).

### Microbial functional distribution

Microbial community function, as a proxy for ecosystem function in the oligotrophic Indian Ocean, was examined via genomic and proteomic annotations based on the OM-RGC dataset (Fig. 2C, D). In the metagenome, genes accounting for 70.5 ± 2.7% and 52.0 ± 2.2% of the reads could be functionally assigned to the cluster of orthologous groups (OGs) and KEGG orthology (KO) functional groups, while these numbers increased to 87.8 ± 1.5% and 69.7 ± 1.8% in the metaproteome, respectively (Supplementary Tables S2, S3). The rarefaction analysis of OGs suggests that the genomic and proteomic compositions are nearly saturated (Supplementary Fig. S1). The functional groups of E (amino acid transport and metabolism), C (energy production and conversion) and J (translation, ribosomal structure and biogenesis) were abundant in both total and active communities. However, the functional groups of G (carbohydrate transport and metabolism), O (post-translational modification and protein turnover) and L (replication, recombination and repair) categories were more abundant in the metaproteome than in metagenome, while F (nucleotide transport and metabolism), H (coenzyme transport and metabolism) and M (cell wall/membrane/envelope biogenesis) were the opposite. Regardless of different abundances among functional groups, their spatial variations, that are represented by coefficient variations (CVs), insignificantly varied in both the metagenomic and metaproteomic datasets. Furthermore, 47% and 52% of all detected OGs accounting for nearly 100% and more than 95% of gene and protein abundances, respectively, were ubiquitous at each site, which were defined as core OGs (Supplementary Fig. S2). In both core and non-core OG subsets, the metaproteome CVs were larger than their counterparts in the metagenome (Fig. 2E), and the same in terms of KO annotation. Overall, microbial metabolic function had a larger spatial variation at the proteomic than at the genomic level.

### Environmental drivers of the community

**Fig. 3:**
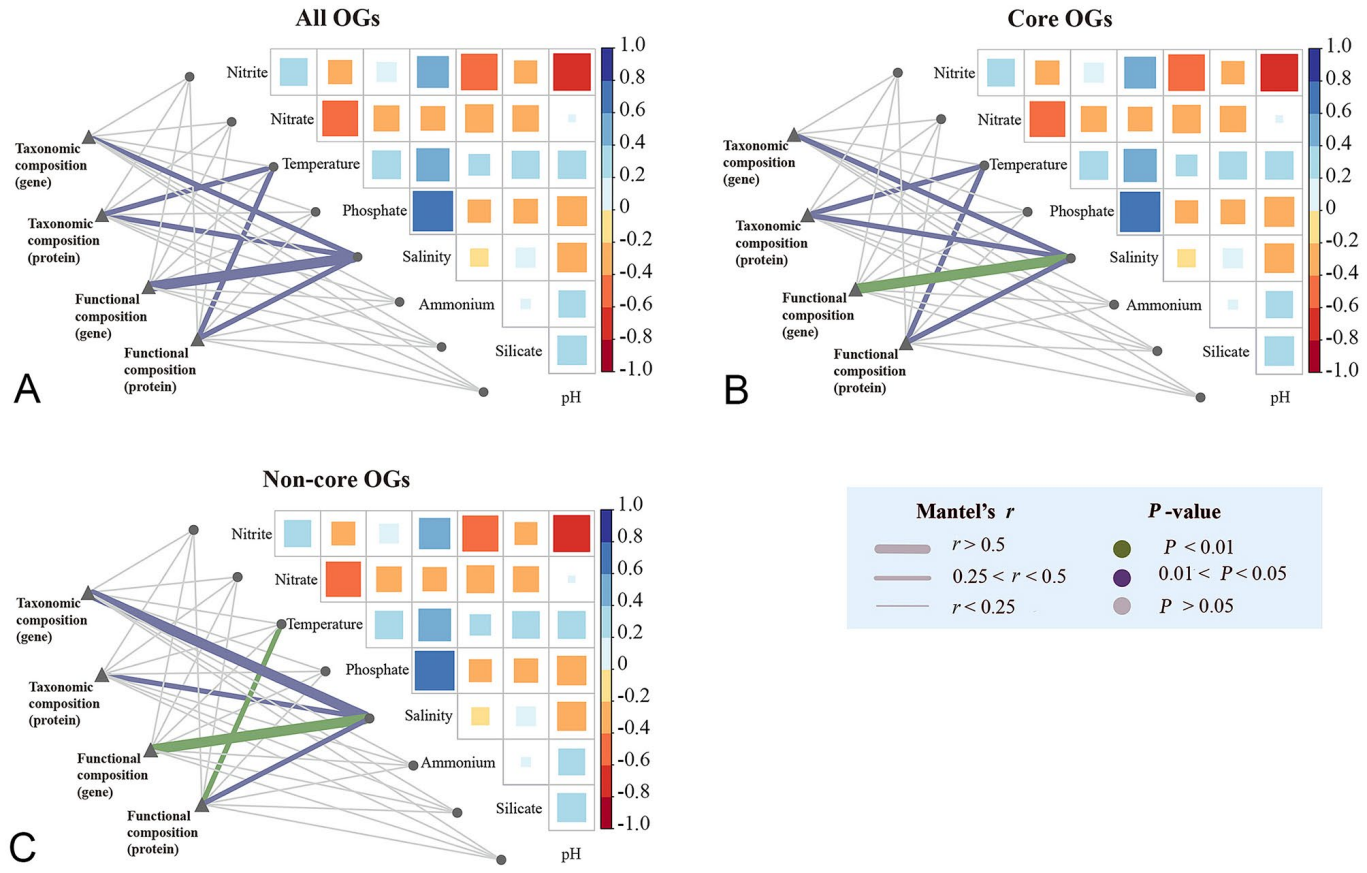
Environmental drivers of genomic and proteomic composition in the oligotrophic Indian Ocean. Different subsets of omic data (**A**: all OGs; **B**: core OGs; **C**: non-core OGs) are used to correlate with the measured environmental factors based on the Mantel tests. The pairwise comparisons of environmental factors only show a significant correlation between salinity and phosphate concentrations. The Mantel’s *r* statistic and statistical significance denoted by edge width and color

**Fig. 4:**
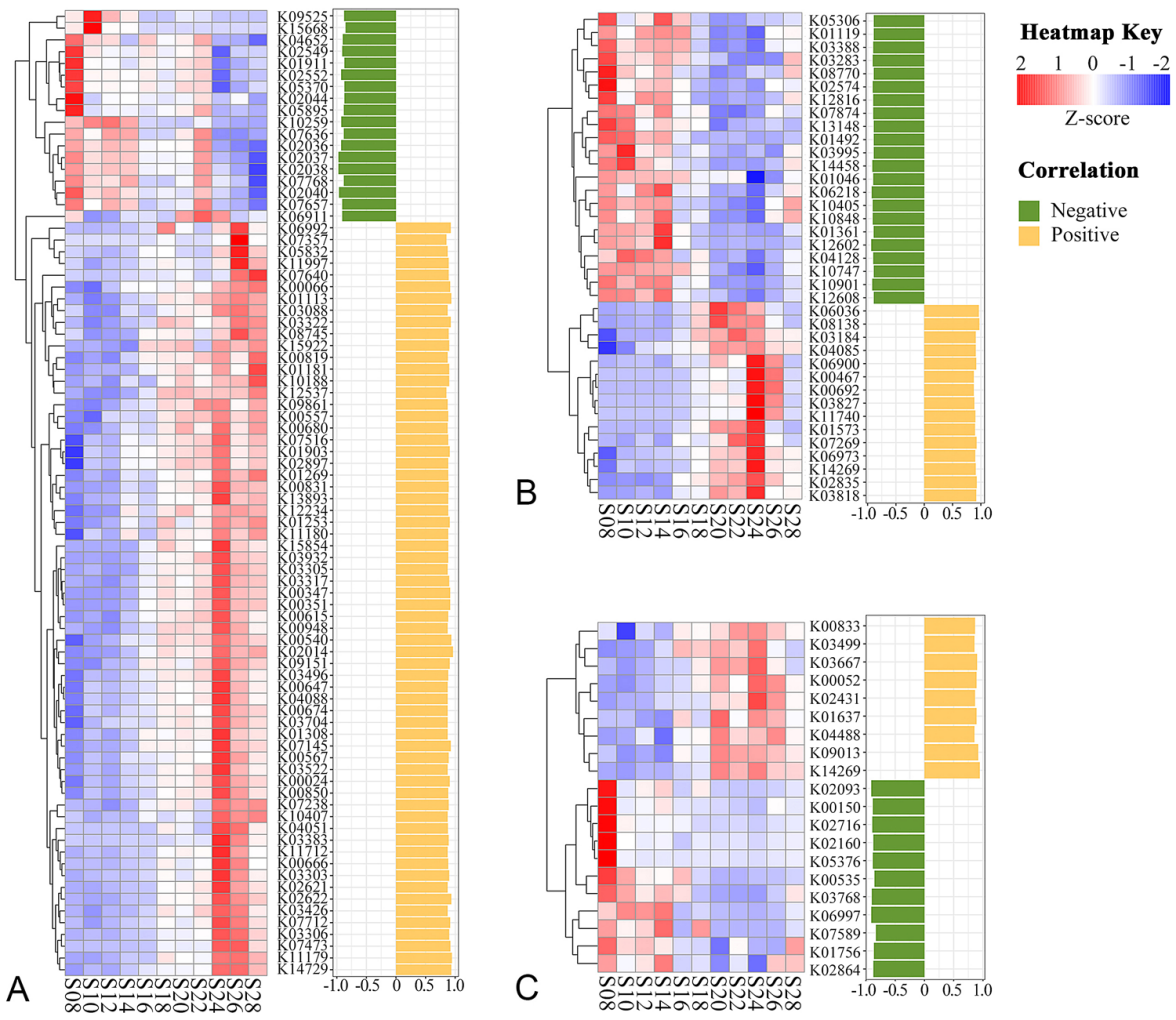
Functional tuning in response to salinity (**A**) and temperature (**B** and **C**). Yellow and green bars indicate KOs positively and negatively correlated with the two environmental factors, respectively. The color gradients denote the *Z* score normalized relative abundance of KOs

The low concentration of nutrients at all sites is consistent with the feature of oligotrophic oceans. The temperature ranged between 29 °C and 32 °C, with the sites closer to the equator having a higher temperature (Fig. 1B). Salinity decreased across all sites from west to east (Fig. 1C). The pairwise correlation between environmental factors suggested that most of these factors were independent, except for salinity which was positively correlated with phosphate concentration (Fig. 3). The Mantel test analysis indicated that both genomic and proteomic compositions were significantly (*P* < 0.05) correlated with salinity, regardless of the taxonomic and functional compositions, while the metaproteomic variations across sites were associated with temperature (Fig. 3A). When only considering the subset of core or non-core OGs, the pattern remained almost unchanged (Fig. 3B, C). A Spearman correlation analysis using mapped KOs revealed that 82 and 37 KOs from the metagenome were significantly correlated with salinity and temperature, respectively, while in their counterparts in the metaproteome there were only one and 20 KOs (Fig. 4). Except for the four KOs that correlated with pH, silicate and phosphate concentrations, none was significantly associated with other environmental factors (Supplementary Table S4), confirming that salinity and temperature were the most significant environmental factors controlling the microbial community in the oligotrophic Indian Ocean.

## Discussion

### Stability of the microbial community in the oligotrophic Indian Ocean

The present study investigated both genomic and proteomic compositions of the microbial communities covering a vast area of the surface Indian Ocean (Figs. 1, 2). Spatial variation in the microbial community at the surface has been observed in regions with much more significant changes in environments, such as at the global ocean scale (Sunagawa et al. 2015), across several biogeochemical provinces (Rusch et al. 2007; Zheng et al. 2016), and from coastal to open ocean (Morris et al. 2010; Ortega-Retuerta et al. 2013). The taxonomic composition of the microbial community is much similar in the open ocean, including the Indian Ocean, and is notably less variable at the phylum level (class level for *Proteobacteria*) than at the order level (Sunagawa et al. 2015; Zheng et al. 2016). The taxonomic composition in the surface Indian Ocean was consistently similar with a predominance of *Cyanobacteria*, *Alphaproteobacteria*, and *Gammaproteobacteria* at all sites, but was more dynamic at lower taxonomic levels, such as the genus level (Fig. 2A, B). In contrast to the global ocean microbiome (Sunagawa et al. 2015), the phylum-level (even the genus level) taxonomic variability was not higher than functional variability (OG or KO categories) in either metagenomes or metaproteomes, regardless of the uniform distribution of functional compositions across sites (Fig. 2). This suggests that the Indian Ocean surface microbial community did not exhibit functional redundancy, as was more obvious in the global ocean microbiome, probably owing to the different degrees of environmental changes at different biogeographic scales.

The taxonomic compositions of the total community (metagenome-level) in the surface Indian Ocean was weakly correlated with salinity; whereas the active community (metaproteome level) was linked to both salinity and temperature (Mantel’s *r* < 0.5, *P* < 0.05, Fig. 3). The metagenomic study of the global ocean microbiome has implied that temperature is the main driver of the community taxonomy (Sunagawa et al. 2015). It has also been demonstrated that salinity is the primary environmental control on the taxonomic composition of marine microbial communities, including subtropical regions of the Indian Ocean (Dupont et al. 2014; Fortunato and Crump 2015; Herlemann et al. 2011; Lozupone and Knight 2007; Zhang et al. 2006; Zheng et al. 2016). A recent study has shown that rare taxa in a community might be more sensitive to salinity than their more abundant counterparts (Yang et al. 2016). In the present metagenomic data, 58 bacterial genera that significantly correlated with salinity were of low abundance (Supplementary Fig. S3). The salinity-positively-correlated bacteria included several genera groups of *Alphaproteobacteria*, *Gammaproteobacteria*, *Bacteroidetes*, *Actinobacteria*, *Betaproteobacteria*, and *Firmicutes*. In another study comparing microbial communities from freshwater and marine habitats, it is found that *Alphaproteobacteria*, *Gammaproteobacteria* and *Bacteroidetes* prefer high salinity habitats (Dupont et al. 2014). However, in the metaproteome, 5 and 11 bacterial genera had significant correlations with salinity and temperature and all these genera were rare in the community (Supplementary Fig. S3). These results demonstrate that the dominant groups shape the stable communities of the surface Indian Ocean, regardless of the variations in the abundance of rare groups, which were sensitive to environmental perturbations, including salinity and temperature.

### Functional tuning in response to salinity change

Metagenomic studies have revealed that many functional potentials can shift with salinity (Dupont et al. 2014; Kimbrel et al. 2018). Interestingly, far more KOs were affected by salinity in the metagenome than in the metaproteome (Fig. 4), which is consistent with the results of the Mantel test analysis. Salinity-correlated KOs in the metagenome included those involved in oxidative stress, two-component systems, carbohydrate, and lipid metabolisms (Fig. 4A).

#### Oxidative stress

It was found that salinity increased the abundance of quinone reductase genes, including Na^+^-transporting NADH: ubiquinone oxidoreductase (*Nqr*, K00347 and K00351) and F_420_H_2_-dependent quinone reductase (*Fqr*, K00540), but decreased the abundances of O-succinylbenzoate synthase (K02549), O-succinylbenzoic acid-CoA ligase (K01911) and menaquinone-specific isochorismate synthase (K02552) genes, which are associated with the biosynthesis of an isoprenoid quinone, menaquinone, other than ubiquinone. Consistently, more abundant *Nqr* genes and less abundant menaquinone biosynthesis genes are reported in salty environments (Dupont et al. 2014). This and our studies suggest that high salinity tuned the microbial community, giving preference to ubiquinone as an electron acceptor in quinone reductase. Furthermore, the abundance of the coenzyme F_420_ L-glutamate ligase (*CofE*, K12234) gene, which is involved in F_420_ biosynthesis, increased in the high salinity environment. It is proposed that an *Fqr* specifically uses F_420_H_2_ for the two-electron reduction of endogenous quinone, thereby making it possible to prevent the formation of semiquinone and the resulting superoxide (Gurumurthy et al. 2013). The increase of both *CofE* and *Fqr* with salinity suggests that F_420_H_2_ has an important role in countering oxidative damage in higher salinity environments. The role of other quinone reductases in protecting against oxidative stress has been well documented (Gurumurthy et al. 2013). These results indicate that genes associated with quinone reductases and coenzyme biosynthesis to eliminate reactive oxygen species are regulated by salinity.

The scavenging of several metals, such as iron, manganese and zinc, was highly positively correlated with salinity. These transporters included iron complex outer membrane receptor protein (*CIRA*, K02014), putative ABC transport system permease protein (K05832), manganese transport protein (*MntH*, K03322), and zinc transporter (*ZupT*, K07238). Similarly, the abundance of transporters for iron, manganese and zinc that change with salinity has been reported in previous metagenomic studies (Dupont et al. 2014; Eiler et al. 2014). Unlike the abundance of ABC transporters for the iron complex (low salinity) and Fe^3+^ (high salinity), depending on the salinity (Dupont et al. 2014), merely the abundances of transporters affiliated with the iron complex increased with salinity in this study. Consistently, the high abundance of cytoplasmic iron level-regulating protein *YaaA* (K09861) and heme oxygenase *IsdG*/*IsdI* (K07145) were also observed at high salinity sites. In the cytoplasm, *IsdG*/*IsdI* can degrade exogenous haem to release Fe^2+^ (Reniere and Skaar 2008; Reniere et al. 2010). These results suggest that the microbial cellular iron level is regulated in a salinity-dependent manner via the iron acquisition from the iron complex in the oligotrophic Indian Ocean. In addition, the *IsdG*/*IsdI*-mediated haem degradation facilitates the virulence of a bacterial pathogen (Reniere and Skaar 2008). Bacterial *MntH* is a selective manganese transporter in response to reactive oxygen and pathogenesis (Kehres et al. 2000). In addition, the ions of Fe^2+^, Mn^2+^, or Zn^2+^ are the metal cofactors of cytosolic superoxide dismutase used to detoxify reactive oxygen during pathogenesis, or under other stress conditions (Kehres et al. 2000). The high abundance of metal transporters suggests that the enhancement of metal acquisition in the community might be associated with the microbial response to oxidative stress induced by high salinity.

#### Two-component systems (TCSs)

TCSs are signaling pathways that enable microbial cells to sense, respond and adapt to changing environments and stressors. In the present study, several genes that belong to TCSs were significantly correlated to salinity, including *SenX3* (K07768), *PstS* (K02040), *PhoR* (K07636), *PhoB* (K07657), *PhoD* (K01113), *CpxA* (K07640), *GlnG* (K07712) and *DctR* (K11712). Among them, the membrane-bound sensors of *SenX3* and *PhoR*, and the response regulator *PhoB* involved in phosphate starvation response were more abundant at low salinity sites where phosphate (Pi) concentration was also low. The same pattern was observed in Pi-specific Pst transport system, including substrate-binding protein *PstS*, ATPase *PstC*, transmembrane proteins *PstA* and *PstB*, and Pi-non-specific Phn transport system, such as the substrate-binding protein *PhnD*. All these genes that encode the ABC-type high-affinity Pi transport system are generally induced under Pi-limitation (Gebhard et al. 2006). However, with the abundance of low-affinity Pi transporters, the Pit system (K03306) significantly increased in those sites with high salinity and Pi concentration, which is consistent with the constitutive expression of Pit in a Pi excess environment (Gebhard et al. 2006). Surprisingly, none of these genes was significantly correlated with the Pi concentration in this study. A similar result has also been reported in a previous metagenomic study (Fraser et al. 2018). These results suggest that salinity regulates the phosphorus utilization in the microbial community via the metabolic tuning of Pi-related signals and transport systems.

In the TCS OmpR family, *CpxA* is a sensor histidine kinase that detects a wide variety of envelope stresses, such as misfolded proteins and high osmolality (Weatherspoon-Griffin et al. 2014). The increase of *CpxA* with salinity suggests an adaptation to salt-induced envelope stress. Coincidently, *RpoE* (K03088) was more represented in the western Indian Ocean with higher salinity, in which the function is a sigma factor to direct RNA polymerase for regulating the expression to combat envelope stress (Rhodius et al. 2006).

#### Carbohydrate and lipid metabolisms

The abundance of several genes associated with carbohydrate metabolism significantly increased with salinity. *AlgD* (K00066) functions as the irreversible conversion of GDP-mannose to GDP-mannuronic acid, which is a nucleotide sugar used by bacteria solely for alginate synthesis (Snook et al. 2003). Alginate can absorb water and is an important viscous exopolysaccharide for biofilm formation. The increase in *AlgD* abundance with salinity suggests that biofilm formation from alginate might protect against salt-induced water loss. It has been reported that in natural communities, many carbohydrate-active enzymes in the glycoside hydrolase family can alter their gene abundance with salinity (Kimbrel et al. 2018). In the present study, two genes from this family, *XynA* (K01181) hydrolyzing xylans and *YihQ* (K15922) cleaving sulphoquinovosides from sulfoquinovosyldiacylglycerol, were more represented at high salinity sites. Furthermore, more abundant lactose/arabinose ABC transporter *LacE*/*AraN* (K10188), *PfkA* (K00850) in glycolysis, *TktA*/*TktB* (K00615) and *PrsA* (K00948) in the pentose phosphate pathway were also detected. It appears that carbohydrate metabolism of the community was tuned by salinity. In addition, genes involved in fatty acid transport (K08745), biosynthesis (K00666 and K00647) and beta-oxidation degradation (K14729 and K07516) were more abundant at high salinity sites. It has been reported that microbial lipid composition can be modified through salinity changes (Elkahoui et al. 2004). These results suggest that microbes might be able to alter their fatty acid biosynthesis and degradation in response to ambient salinity variation.

### Functional tuning in response to temperature change

#### Temperature-correlated function potential

In the metagenome, 15 KOs dispersed in diverse pathways were positively correlated with temperature (Fig. 4B). Elevated temperature increased the abundance of aminoalkylphosphonate N-acetyltransferase *PhnO* (K03827) but decreased the abundance of *PhnX* (K05306), that encodes phosphonoacetaldehyde phosphohydrolase. Each participates in different pathways of C-P bond cleavage but PhnO has broader substrate affinities than PhnX, which is restricted to 2-aminoethylphosphonate (Misra et al. 2012; Quinn et al. 2007). These results suggest that a shift of temperature affects the utilization of phosphonate by the community. Cellular respiration might also be stimulated by temperature. For example, high temperature increased the abundance of the *ubiF* (K03184) gene involved in the biosynthesis of ubiquinone, an important component of the electron transport chain in oxidative phosphorylation. Similarly, translation was expected to be enhanced by high temperature, since the gene abundance of a peptide chain release factor *PrfA* (K02835) increased with elevated temperature. In addition, *SacB* (K00692) was more abundant at high-temperature sites, suggesting that the increase in temperature allows more sucrose-sensitive *SacB*-containing microbes to survive, since its expression is lethal to sucrose-sensitive bacteria, while high temperature can alleviate this sensitivity (Blomfield et al. 1991). Furthermore, it is proposed that N4 bacteriophages require *NfrB* (K11740) for irreversible adsorption and phage genome injection through the host inner membrane (Kiino and Rothman-Denes 1989). Increasing abundance of *NfrB* indicates that temperature might enhance bacteriophage infection.

Some KOs in the metagenome were found to be negatively correlated to temperature (Fig. 4B). The gene abundance of *TGL2* (K01046) and *MGAT* (K14458) decreased at high-temperature sites, indicating the presence of community temperature-regulation of phospholipid synthesis, since the hydrolysis of triacylglycerol through the lipase *TGL2* and *MGAT* in the monoacylglycerol pathway can generate diacylglycerol, a precursor of phospholipids. Interestingly, the low abundance of *HdrA2* (K03388) was associated with high temperature. It has been demonstrated that *HdrA2* plays a specific role in anaerobic methane production in acetotrophic and methylotrophic methanogens (Yan et al. 2017). The anoxic aggregate microenvironment hypothesis may explain the occurrence of HdrA2-mediated methanogenesis in the oxic surface ocean (Reeburgh 2007). Nevertheless, our results imply the presence of temperature-driven methanogens in the surface ocean. In addition, a number of temperature-negative-correlated genes associated with nucleotide metabolism (K01119 and K01492), eukaryotic type DNA recombination and excision repair (K10848 and K10747), spliceosome (K03283, K12816, and K13148) and mRNA degradation (K12602, K12608, and K06218) were also found, many of them originating from eukaryotic algae, suggesting more frequent and complex modifications of cellular processes at genetic and transcriptional levels, particularly in eukaryotic algae, under high-temperature stress. In addition, decreases in ubiquitin C (K08770) and *AprE* (K01361) and an increase in Zn-dependent membrane protease *YugP* (K06973) imply the involvement of temperature in ubiquitylation and protease-mediated post-translational modifications.

#### Temperature-correlated function expression

In contrast to the metagenome, completely different functions represented by the 20 KOs in the metaproteome were selected when correlating with temperature (Fig. 4C). Among these KOs, almost half were upregulated at high-temperature sites, including stress response, proteolysis, carbon metabolism, nitrogen fixation, K^+^ hemostasis, and vitamin biosynthesis. At heat-shock temperatures, HslU ATPase (K03667) in energy-dependent proteolytic machines denatures protein substrates and translocates the unfolded polypeptide into HslV peptidase for protein degradation (Burton et al. 2005). The observation of a more abundant HslU protein under higher temperature is consistent with the HslUV-mediated heat-shock response (Burton et al. 2005). The elevated temperature might cause oxidative stress. It is speculated that SufC (K09013) is a key component of the *suf* system involved in the protection, activation and repair of damaged iron–sulfur [Fe–S] proteins under oxidative stress (Johnson et al. 2005; Nachin et al. 2003). The [Fe–S] cluster is ubiquitous and functionally versatile in a wide range of enzymes involved in many cellular processes (Johnson et al. 2005). An increasing expression of *sufC* with temperature might be an important microbial adaptation to high temperature via protecting [Fe–S] proteins against oxidative damage. In addition, bacterial intracellular K^+^ hemostasis is largely mediated by specialized K^+^ transporters, in which the gate of the K^+^ channel is controlled by TrkA (K03499) (Cao et al. 2013). The increased abundance of TrkA protein under high temperature implies that TrkA is involved in K^+^ hemostasis regulation.

Our results also indicate that a few biosynthetic activities in the microbial community were affected by temperature. A key enzyme splitting isocitrate in the glyoxylate cycle, AceA (K03667), increased in abundance with increasing temperature, suggesting that carbon metabolism is tuned by temperature. By bypassing the steps of carbon dioxide production in the TCA cycle, the enhanced glyoxylate shunt enables a more economical use of carbon. For nitrogen metabolism, NifU (K04488), a scaffold protein destined for nitrogenase maturation (Johnson et al. 2005), was more abundant at high-temperature sites, suggesting that nitrogen fixation activity might be linked to temperature. Furthermore, the increase of a critical enzyme involved in biotin biosynthesis, BioA (K00833), indicates that a more active biotin biosynthesis is present in the community living in high-temperature waters.

The remaining 11 KOs, such as YggS (K06997), AccB (K02160) and PpiB (K03768), were downregulated in abundance when subjected to high temperature. A study has demonstrated that YggS-deficient cells accumulate 2-ketobutyrate and significantly increase the expressions of enzymes related to isoleucine and valine metabolism (Ito et al. 2013). In contrast to the decreasing abundance of YggS protein, an increasing abundance of LeuB (K00052), which catalyzes the production of 2-ketobutyrate, a precursor of isoleucine, leucine and valine, was found, and this is consistent with the conclusion that YggS-regulation is present in the metabolism of these amino acids (Ito et al. 2013). Temperature might affect the biosynthesis of isoleucine, leucine, and valine via regulating *yggS* gene expression. In addition to the amino acid biosynthesis affected by temperature, the abundance of AccB (K02160), involved in the initiation of fatty acid biosynthesis, decreased in the metaproteome, suggesting the downregulation of fatty acid biosynthesis at high temperatures. Furthermore, the abundance of photosystem II oxygen-evolving enhancer protein PsbO (K02716) and antenna proteins CpeA (K05376) and ApcB (K02093) decreased with increasing temperature, indicating a downregulation of photosynthetic activity caused by high temperature. Moreover, a low abundance of peptidyl-prolyl isomerase PpiB (K03768) was detected at high-temperature sites, which is expected as PpiB is required for optimal bacterial growth at low temperatures (Soderberg and Cianciotto 2008).

### Decoupling between metabolic potential and metabolic activity

**Fig. 5:**
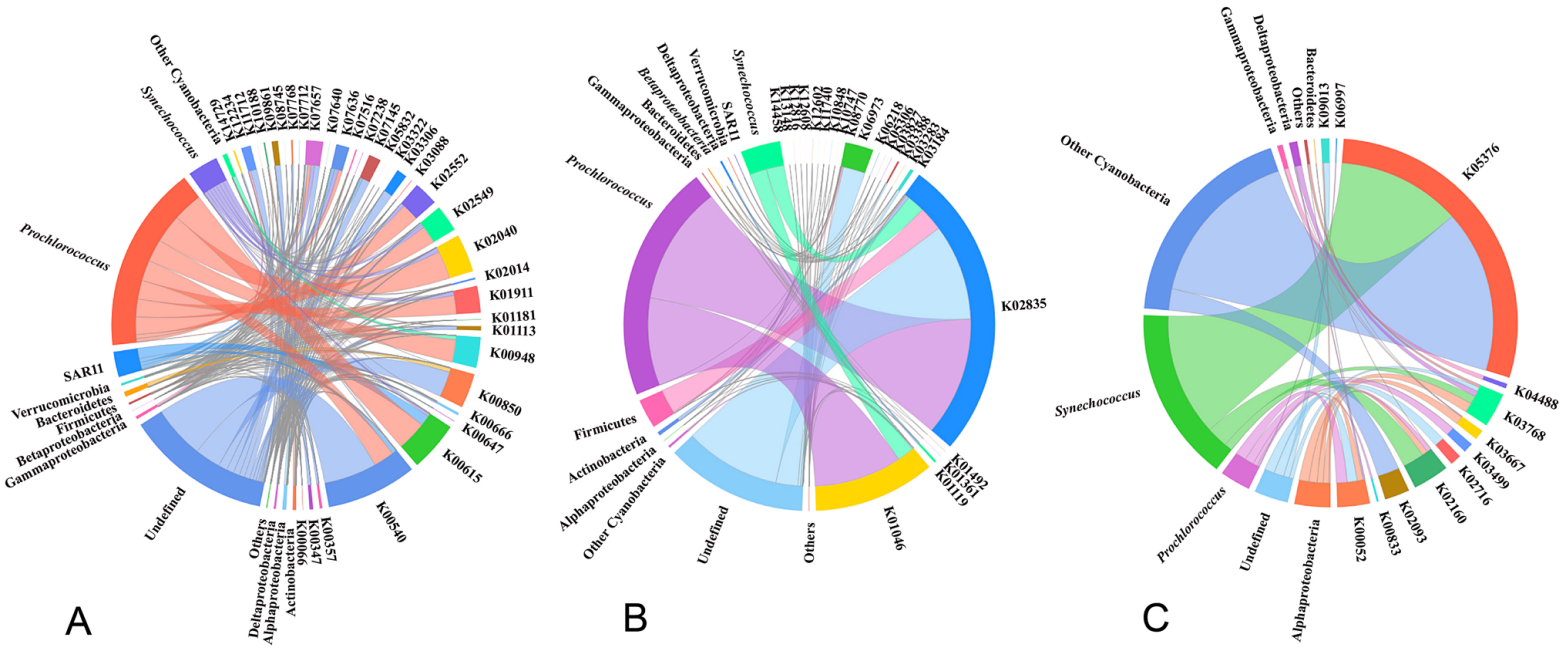
Taxonomic composition of KOs. KOs in the metagenome significantly correlated with salinity (**A**) and temperature (**B**). KOs in the metaproteome significantly correlated with temperature (**C**)

Our integrated meta-omic data demonstrated that several metabolic functions did change with salinity and temperature, regardless of the stable community composition in this region. Furthermore, the genomic composition of the community had less spatial variation, when compared with the proteomic counterpart (Fig. 2E), suggesting that the metabolic adjustments of the microbial community in response to environmental perturbation in the oligotrophic Indian Ocean might have stronger signals at the dynamic gene expression level, when compared with the more static gene context level. The correlation analysis revealed that the same environmental factors were associated with different functions in the metagenomic and metaproteomic-determined compositions of the community, and a significant difference in salinity-correlated KO numbers (1 vs*.* 82) between the metaproteome and metagenome were observed. This demonstrates the significant tuning divergence in metabolic activity and metabolic potential: that is, they decouple from each other to some extent. It is common to observe flexibility of metabolic activity in an organism, regardless of whether they have a constant genome, which is reasonable expectation as different functions between the proteome and genome are selected by an environmental factor at a specific time. However, at the community level, it was found that many environment-selected KOs in the metagenome and metaproteome were derived from diverse and low abundant taxonomic lineages and only a few were affiliated with abundant microbial groups, such as *Prochlorococcus* and *Synechococcus* (Fig. 5 and Supplementary Table S5). This hints that the decoupling is associated with the taxonomic composition, especially with rare bacteria. Nonetheless, even with the tuning divergence between metabolic activity and metabolic potential, both play important roles in maintaining a functional and compositional (phylum level) stable community in oligotrophic oceans.

## Limitations

It should be pointed out that due to limited time for sampling only one sample was taken from each site. This lack of replication will have introduced a level of uncertainty. In particular, whether there is more variation at site S8 than at other sites remains unknown without replicates. Therefore, to minimize the uncertainty caused by the lack of replication, both metagenomic and metaproteomic analyses were conducted and the results show that the dominant taxa at site S8, *Synechococcus,* was responsible for most of the variation in microbial community composition. This observation is consistent with site S8 being close to the coast where *Synechococcus* is the usually the dominant cyanobacterial group (Karlusich et al. 2020; Wang et al. 2021). Interestingly, a recent study has shown that true replication is not always possible in oceanography, because the ocean is a fluid environment and ‘replicate’ samples from different water mass might actually be collected even within a short time (Saito et al. 2019). Therefore, when replication is not possible, consistency in trends across vertical or horizontal structures is often used as an alternative approach by oceanographers. Our study reflected the trend of stable structure in microbial community across the north Indian Ocean. Of course, sufficient biological replicates, combined with multi-omics analyses are encouraged to reduce the risk of incorrect interpretations caused using a single sample.

## Conclusion

In this study, metagenomic and metaproteomic approaches were integrated to answer how the microbial community across a large area of an oligotrophic ocean performed, in terms of composition and function, in response to environmental perturbation. A similar pattern among sites, in terms of both genomic and proteomic compositions, suggested that both total and active communities were relatively stable. Regardless of the slight environmental fluctuations, salinity and temperature could still be the main drivers of total and active communities, respectively. Dominant bacteria, such as *Prochlorococcus*, *Synechococcus*, and SAR11, shaped the stable community, while rare taxa were environment sensitive.

Interestingly, metabolic tuning occurred in both function potential and activity in response to salinity and temperature changes, but they decoupled. Metabolic variabilities in the microbial community at different molecular levels were demonstrated by the present metagenomic and metaproteomic data, suggesting that this approach could provide comprehensive insights into the metabolic activities of the microbial community and their interactions with ambient environments, and that metabolic tuning was one of the important mechanisms to sustain the stability of the microbial community in the oligotrophic ocean. Owing to the potential biases caused by a single meta-omic survey, more attempts should be devoted to conducting in situ integrated meta-omic studies via connecting different meta-omics data, to comprehensively understand the microbial community metabolism. This is important for the mechanistic explanation of the metabolic status of an oceanic region, as well as the microbial interactions with their ambient environments.

## Materials and methods

### In situ sampling

The samples were collected from a depth of 5-m during the Indian Ocean leg of the 26th Chinese COMRA cruise of the R/V Dayang Yihao in May 2012. A total of 11 microbial communities were sampled from 11 sites (Fig. 1A). Seawater sample of 200–900 L for each site was sequentially filtered through GF/A (1.6 µm, 142 mm filter diameter, Whatman) and Supor polyethersulfone (0.2 µm, 142 mm filter diameter, Pall) membrane filters during the daytime. Once collected, the Supor filters were flash frozen in liquid nitrogen, and stored at − 80 °C until DNA and protein extractions in an indoor laboratory. The microbial samples collected on the Supor filters at each site were equally separated before DNA and protein extractions. Environmental parameters (Fig. 1B–I) such as temperature, salinity and pH were monitored using a SBE 21 SeaCAT Thermosalinograph (Seabird Co.) during the cruise, while the concentrations of nutrients, including phosphate, silicate, ammonia, nitrite and nitrate were measured in the laboratory using spectrophotometry methods of the ascorbic acid–molybdenum blue, molybdosilicate blue, sodium hypobromite oxidation, diazo–azo reaction, and zinc–cadmium reduction as described in the practical guidelines of seawater analysis (Hansen and Koroleff 1999) and the “Specification for oceanographic survey” (GB/T 12763.4-2007).

### Metagenomics

The metagenomic experiments were performed in a similar way to previous studies (Fang et al. 2018; Huang et al. 2017). In brief, DNA was extracted using a PowerSoil^®^ DNA Isolation Kit (USA), and fragmented using a Covaris E220 focused-ultrasonicator (Covaris, UK), according to the manufacturer’s instructions. DNA fragments between 400 and 700 bp were selected for a single-strand circular DNA library construction for each sample. The barcoded libraries were pooled and sequenced according to the BGISEQ-500 protocol (SOP AO) employing the SE50 mode (Huang et al. 2017).

Raw FASTQ reads were quality evaluated and trimmed using SOAPnuke (v1.5.3) to generate high-quality reads (Chen et al. 2018). To identify the microbial functional genes, all high-quality reads were aligned against the global Ocean Microbial Reference Gene Catalog (OM-RGC) dataset containing > 40 million nonredundant representative genes of marine microbiome (Sunagawa et al. 2015) using bowtie2 (Version 2.2.6) (Langmead and Salzberg 2012) with parameters “–sensitive –mp 1,1 –np 1 –score-min L,0, −0.1”. Gene relative abundance was calculated using the number of uniquely mapped reads divided by the length of the gene, according to a previous study (Qin et al. 2012). Taxonomic and functional annotations were extracted from the original gene’s information based on the OM-RGC dataset. Taxonomic and functional (KO or OG) abundances were calculated by summing up the relative abundances of individual genes assigned to the same group.

All metagenomic raw reads were submitted to in the CNSA (https://db.cngb.org/cnsa/) of CNGBdb with accession code CNP0000411.

### Metaproteomics

Each frozen 0.2 µm filter was ground into powder with the aid of liquid nitrogen and transferred to a new tube. For the per gram powder, 20 ml of TRIzol^®^ (Molecular Research Center, Inc.) was added. Then, the blended mixture was sonicated on ice (100 w, eight seconds on and 16 s off, for 60 cycles) and incubated at room temperature for 1 h. Afterward, the supernatant was transferred to a new tube after centrifugation at 13,500 *g* for 40 min at 4 °C, and combined with 0.2 ml of chloroform per ml of starting TRIzol volume. The mixture was vortexed for 15 s and rested for 5 min at room temperature for phase separation. After centrifugation, the bottom phenol layer was carefully transferred and combined with 0.3 ml ethanol per ml of starting TRIzol volume. To remove the non-proteinaceous material, such as DNA, the solution was mixed by inversion and incubated for 10 min, followed by a centrifugation step at 2000 *g* for 5 min at 4 °C. Then, the supernatant was transferred to a new tube, and the proteins were precipitated overnight at − 20 °C using isopropanol. Afterward, the proteins were pelleted via centrifugation at 12,000 *g* for 30 min, resolubilized in buffer containing 7 mol/L urea, 2 mol/L thiourea, and CHAPS (4% w/v), and stored at − 80 °C.

Protein concentrations were tested using a 2D Quant kit (GE Healthcare, USA). For each sample, 100 µg of protein was loaded onto a 30 k Microcon filtration device (Millipore) for the following filter-aided sample preparation (Wisniewski et al. 2009). The protein buffer solution on the device was replaced and rinsed three times using another buffer (8 mol/L Urea in 0.1 mol/L Tris/HCl, pH 8.5), followed by dithiothreitol reduction and iodoacetamide alkylation steps. Then, the protein mixture was subjected to trypsin (Promega, USA) digestion at 37 ℃, twice. Afterward, the collected peptide solution was desalted and vacuum dried. The peptides were reconstituted with buffer A (5% acetonitrile [ACN] and adjusted to 9.8 with ammonia) to 2 ml and loaded onto a high pH Gemini C18 column (5 µm, 4.6 × 250 mm, Phenomenex) equipped on a Shimadzu LC-20AB HPLC system for separation. During the elution, the flow rate was 1 ml/min and the gradients for buffer B (95% ACN, pH 9.8) were set as follows: 5% for 10 min, 5–35% of linear gradient for 40 min, 35–95% of linear gradient for 1 min, 95% for 3 min, decreasing to 5% for 1 min, and finally, equilibrating at 5% for 10 min before the next injection. Absorbance at 214 nm was used to monitor the peptide concentration. Peptide fractions were collected at each minute, pooled into 20 fractions at a similar intensity, and vacuum dried.

Each fraction was resuspended in buffer C (5% ACN, 0.1% formic acid [FA]) and diluted to approximately 0.5 μg/μl. After centrifugation at 20,000 *g* for 10 min, 10 μl of the supernatant was automatically loaded on a 2 cm C18 trap column equipped on a LC-20AD nano HPLC (Shimadzu, Kyoto, Japan). The trapped peptides were transferred onto an analytical C18 column (75 μm, i.d. × 10 cm) packed in-house at a flow rate of 8 μl/min for 4 min. The elution was run at 300 nl/min with a gradient of buffer D (95% ACN, 0.1% FA), as follows: 5% for 5 min, 5–35% for 35 min, 35–60% for 5 min, 60–80% for 2 min, 80% for 4 min, returning to 5% for 1 min, and equilibrating at 5% for 10 min before the next injection. Data acquisition was performed on a Triple TOF 5600 mass spectrometer (AB SCIEX, Canada) fitted with a Nanospray III source and a pulled quartz tip as the emitter (New Objectives, USA). The instrument operation conditions were as follows: ion spray voltage of 2.5 kV, curtain gas of 30 psi, nebulizer gas of 15 psi, and interface heater temperature of 150 ℃. The TOF mass spectrometry (MS) scans were operated in reflection mode at a resolution of at least 30,000. In the information-dependent acquisition mode, MS survey scans in a mass range of 350–1,250 were acquired at 100 ms, followed by 40 MS/MS scans of an MS peak intensity of above 150 counts per second and a charge between +2 and +5. Total cycle time was fixed to 2.8 s. Dynamic exclusion was set for 15 s.

Raw peptide data (.wiff) were converted to the Mascot generic file format (.mgf) using the SCIEX MS Data Converter (version 1.3 beta). A three-step search strategy, which was previously described (Zhang et al. 2016), was used to search against a subset of the OM-RGC database derived from the SE50 sequencing. In brief, the X!Tandem (2017.2.1 version) without any criterions was used for the first search step. Sequences from the search results were extracted and combined with its decoy datasets as a transition database for the second X!Tandem search with more stringent filtrations. The secondary search results were constructed as the final library. Finally, MaxQuant (1.6.1.0 version) was used to search against the last library for identification and quantification. Parameters for the first two X!Tandem search steps were set, as follows: the mass tolerance for intact peptides and fragmented ions were 0.07 Da and 40 ppm, respectively; the enzyme was restricted as trypsin, allowing one missed cleavage; the variable modification included Gln- > pyro-Glu (N-term !), oxidation (M) and deamidation (NQ), while carbamidomethyl cysteine was the fixed modification; the charge states were set to + 2 and + 3. The second search was different from the first one and was a decoy dataset used for the false discovery rate (FDR) filtration. The proteins were grouped when identified by the same set or subset of peptides. A 1% cutoff for FDR was set both on the spectrum and protein levels. Protein or protein groups were finally accepted with the more stringent criterions of at least one peptide and two spectra, to increase the confidence of the identification. For protein semi-quantification, the ion peak intensity of the peptides in a protein group was summed up. For consistency considerations between metagenomics and metaproteomics, taxonomic and functional annotations were also based on the OM-RGC annotation.

The Maxquant results and MS raw files were deposited to the ProteomeXchange Consortium (http://www.proteomexchange.org) with dataset identifier PXD016285.

## Statistical analysis

All the statistical analyses were performed in R (v. 3.5.1). The Mantel test was run following the instruction of vegan (2.5-4) R package to determine correlations between environmental factors and the microbial community in terms of both taxonomic and functional compositions. Data from the S10 site were removed and not considered in the Mantel test due to missing environmental nutrient parameter values at this site. A Euclidean distance method was calculated using the *z* score transformed environmental parameters, while a Bray–Curtis distance method was used to calculate the matrices of taxonomic and functional compositions. Calculations of the correlation coefficients between matrices of environments and compositions were followed using the permutation test.

To exactly determine which taxonomic and functional features in the community were affected by environmental factors, the Spearman’s rank correlation test was performed. The Benjamini–Hochberg correction was used to correct the values of multiple tests. Finally, a cutoff of *q* value < 0.1 was applied to determine the significant correlation between community feature and environmental factor. Noted that the missing nutrient data at the S10 site, data of this site were not used when testing the correlation between community feature and environmental nutrient.

## Supplementary Information

Below is the link to the electronic supplementary material.

Supplementary file1 (XLSX 355 KB)

Supplementary file2 (DOCX 2434 KB)
