# Supplementary material for: Metabolic tuning of a stable microbial community in the surface oligotrophic Indian Ocean revealed by integrated meta-omics
Source: Mar Life Sci Technol. 2022 Jan 1;4(2):277–90. doi: 10.1007/s42995-021-00119-6 (PMC10077294; doi:10.1007/s42995-021-00119-6)

Marine Life Science & Technology

**Metabolic tuning of a stable microbial community in the surface oligotrophic Indian Ocean revealed by integrated meta-omics**

Zhang-Xian Xie^1, 2, 3^, Ke-Qiang Yan^4, 5^, Ling-Fen Kong^1^, Ying-Bao Gai^6, 7^, Tao Jin^4^, Yan-Bin He^4^, Ya-Yu Wang^4^, Feng Chen^8^, Lin Lin^1, 3^, Zhi-Long Lin^4^, Hong-Kai Xu^4，5^, Zong-Ze Shao^6, 7^, Si-Qi Liu^4^#, Da-Zhi Wang^1, 3^#

^1^State Key Laboratory of Marine Environmental Science/College of the Environment and Ecology, Xiamen University, Xiamen 361005, China

^2^College of Ocean and Earth Sciences, Xiamen University, Xiamen 361005, China

^3^Southern Marine Science and Engineering Guangdong Laboratory (Zhuhai), Sun Yat-Sen University, Zhuhai, 519082, China

^4^BGI-Shenzhen, Beishan Industrial Zone 11^th^ Building, Shenzhen 518083, China

^5^BGI Education Center, University of Chinese Academy of Sciences, Shenzhen 518083, China

^6^Key Laboratory of Marine Genetic Resources, Third Institute of Oceanography, Ministry of Natural Resources of China, Xiamen 361005, China

^7^State Key Laboratory Breeding Base of Marine Genetic Resources/Fujian Key Laboratory of Marine Genetic Resources, Xiamen 361005, China

^8^Institute of Marine and Environmental Technology, University of Maryland Center for Environmental Science, Baltimore, MD, USA.

#Address correspondence to Si-Qi Liu, siqiliu@genomics.cn or Da-Zhi Wang, dzwang@xmu.edu.cn.

Zhang-Xian Xie and Ke-Qiang Yan contributed equally to this work.

Supplementary Table S1 Metadata for 11 sampling sites across the surface tropic Indian Ocean in this study.

| Category | S8 | S10 | S12 | S14 | S16 | S18 | S20 | S22 | S24 | S26 | S28 |
| --- | --- | --- | --- | --- | --- | --- | --- | --- | --- | --- | --- |
| Latitude | 5°54.3′N | 5°00.4′N | 4°09.9′N | 3°18.0′N | 2°17.8′N | 1°42.8′N | 3°03.1′N | 3°42.0′N | 9°24.1′N | 13°26.3′N | 13°37.1′N |
| Longitude | 93°49.2′E | 89°33.8′E | 85°31.8′E | 81°31.2′E | 76°45.1′E | 72°52.2′E | 66°36.8′E | 63°49.0′E | 59°49.2′E | 56°05.9′E | 54°14.9′E |
| Date, dd/mm/yy | 04/05/12 | 05/05/12 | 06/05/12 | 07/05/12 | 08/05/12 | 09/05/12 | 10/05/12 | 15/05/12 | 17/05/12 | 18/05/12 | 19/05/12 |
| Temperature, ℃ | 29.5 | 29.8 | 29.8 | 29.7 | 30.5 | 30.4 | 31.5 | 31.1 | 31.1 | 30.5 | 30.3 |
| Salinity | 33.7 | 34.1 | 34.2 | 34.2 | 34.7 | 34.9 | 34.8 | 34.6 | 35.8 | 36.0 | 36.2 |
| pH | 8.16 | 8.16 | 8.16 | 8.17 | 8.19 | 8.20 | 8.19 | 8.18 | 8.16 | 8.15 | 8.14 |
| NO_3_^-^, µmol/L | 0.123 | - | 0.252 | 0.272 | 0.123 | 0.262 | 0.063 | 0.034 | 0.163 | 0.024 | 0.163 |
| NO_2_^-^, µmol/L | 0.0483 | - | 0.0282 | 0.0483 | 0.0148 | 0.0483 | 0.0349 | 0.0080 | 0.0550 | 0.0483 | 0.0617 |
| NH_4_^+^, µmol/L | 0.226 | - | 0.301 | 0.386 | 0.450 | 0.173 | 0.333 | 0.354 | 0.290 | 0.386 | 0.173 |
| PO_4_^3-^, µmol/L | 0.067 | - | 0.129 | 0.097 | 0.137 | 0.110 | 0.183 | 0.185 | 0.141 | 0.174 | 0.336 |
| SiO_3_^2-^, µmol/L | 1.17 | - | 0.89 | 0.06 | 1.63 | 3.11 | 0.70 | 2.56 | 2.09 | 2.74 | 0.00 |

Note that nutrient samples for S10 station were lost.

Supplementary Table S2 Single-end 50bp (SE50) sequencing statistics for metagenome of 11 sampling sites across the surface tropic Indian Ocean.

| Category | S8 | S10 | S12 | S14 | S16 | S18 | S20 | S22 | S24 | S26 | S28 |
| --- | --- | --- | --- | --- | --- | --- | --- | --- | --- | --- | --- |
| Raw reads | 816467631 | 775008262 | 848934869 | 810981407 | 859026632 | 725748375 | 766350233 | 773937825 | 755548937 | 739551991 | 730689666 |
| Clean reads | 812822491 | 769172563 | 842361418 | 804349906 | 849940678 | 719349080 | 756203703 | 772367729 | 751145928 | 733421960 | 725164703 |
| Reads matching OM-RGC (%) | 70.8 | 73.4 | 73.9 | 74.0 | 75.4 | 74.9 | 76.7 | 76.0 | 68.8 | 68.0 | 73.2 |
| Genes matching OM-RGC | 3463085 | 2139604 | 2773705 | 3193747 | 3178592 | 2963542 | 3639214 | 2899695 | 2276371 | 2875357 | 3888760 |
| Genes assigned to OG (%) | 60.5 | 64.6 | 62.2 | 62.9 | 63.5 | 64.1 | 61.7 | 63.5 | 66.1 | 64.0 | 62.4 |
| Genes assigned to COG (%) | 51.1 | 55.3 | 53.1 | 53.8 | 54.4 | 54.8 | 52.7 | 54.4 | 57.0 | 54.7 | 53.3 |
| Genes assigned to KEGG (%) | 41.8 | 45.7 | 43.8 | 44.3 | 44.7 | 45.0 | 43.3 | 44.7 | 46.8 | 44.9 | 43.9 |
| Genes from Bacteria (%) | 60.0 | 64.8 | 62.7 | 63.4 | 64.6 | 65.2 | 63.0 | 65.4 | 67.8 | 64.7 | 63.0 |
| Genes from Archaea (%) | 1.3 | 1.1 | 1.1 | 1.1 | 1.1 | 0.9 | 0.9 | 0.9 | 0.9 | 1.2 | 1.2 |
| Genes from Eukaryotes (%) | 2.9 | 1.6 | 1.7 | 1.8 | 1.7 | 1.5 | 1.6 | 1.6 | 1.5 | 1.7 | 1.8 |
| Genes from Viruses (%) | 10.3 | 10.1 | 10.1 | 9.8 | 8.7 | 9.4 | 9.7 | 8.6 | 8.7 | 9.8 | 9.6 |
| Genes from unclassified (%) | 25.5 | 22.5 | 24.4 | 23.9 | 23.9 | 23.0 | 24.7 | 23.6 | 21.2 | 22.7 | 24.4 |

Supplementary Table S3 Protein statistics for metaproteome of 11 sampling sites across the surface tropic Indian Ocean.

| Category | S8 | S10 | S12 | S14 | S16 | S18 | S20 | S22 | S24 | S26 | S28 | average | SD |
| --- | --- | --- | --- | --- | --- | --- | --- | --- | --- | --- | --- | --- | --- |
| Total spectra | 367746 | 294212 | 272651 | 308919 | 242734 | 300737 | 253456 | 300882 | 274011 | 300943 | 292555 | 291713 | 32976 |
| Identified spectra (%) | 14.52 | 13.52 | 16.40 | 17.07 | 19.92 | 17.19 | 18.51 | 15.61 | 18.43 | 18.65 | 17.72 | 17.05 | 1.91 |
| Identified peptides | 31512 | 23391 | 25343 | 30199 | 26235 | 28394 | 26793 | 26648 | 27950 | 30673 | 28893 | 27821 | 2442 |
| Peptides for quantifying (%) | 92.07 | 94.65 | 93.90 | 93.10 | 95.22 | 93.86 | 94.30 | 93.90 | 94.02 | 93.81 | 94.25 | 93.92 | 0.81 |
| Proteins being identified | 5991 | 4990 | 5289 | 5879 | 5419 | 5733 | 5453 | 5472 | 5647 | 6075 | 5889 | 5622 | 331 |
| Proteins assigned to OG (%) | 86.9 | 86.9 | 86.9 | 86.5 | 86.6 | 87.2 | 87.3 | 87.9 | 87.6 | 87.5 | 87.6 | 87.2 | 0.4 |
| Proteins assigned to COG (%) | 76.4 | 76.3 | 77.0 | 76.5 | 76.4 | 77.5 | 77.1 | 78.1 | 78.1 | 77.8 | 77.6 | 77.2 | 0.7 |
| Proteins assigned to KEGG (%) | 67.1 | 67.2 | 67.7 | 67.0 | 66.8 | 67.9 | 67.3 | 68.6 | 68.1 | 67.8 | 67.1 | 67.5 | 0.6 |
| Proteins from Bacteria (%) | 89.4 | 89.2 | 89.0 | 88.9 | 88.8 | 89.7 | 89.7 | 89.9 | 90.1 | 89.8 | 89.5 | 89.4 | 0.4 |
| Proteins from Archaea (%) | 0.58 | 0.58 | 0.59 | 0.58 | 0.52 | 0.44 | 0.40 | 0.53 | 0.44 | 0.48 | 0.49 | 0.51 | 0.07 |
| Proteins from Eukaryotes (%) | 1.84 | 1.94 | 1.99 | 1.84 | 1.86 | 1.71 | 1.54 | 1.81 | 1.66 | 1.56 | 1.70 | 1.77 | 0.15 |
| Proteins from Viruses (%) | 1.87 | 2.22 | 2.08 | 2.01 | 2.01 | 2.01 | 1.85 | 1.92 | 1.75 | 1.84 | 2.00 | 1.96 | 0.13 |
| Proteins from unclassified (%) | 6.29 | 6.05 | 6.35 | 6.72 | 6.85 | 6.12 | 6.51 | 5.81 | 6.04 | 6.32 | 6.35 | 6.31 | 0.30 |

Supplementary Table S4 No. of KOs in metagenomic or metaproteomic datasets that are significantly correlated with environmental factors based on Spearman correlation analysis.

| Dataset | Salinity | Temperature（℃） | PO_4_^3-^ | pH | SiO_3_^2-^ | NH_4_^+^ | NO_2_^-^ | NO_3_^-^ |
| --- | --- | --- | --- | --- | --- | --- | --- | --- |
| Metagenomes | 82 | 37 | 2 | 0 | 0 | 0 | 0 | 0 |
| Metaproteomes | 1 | 20 | 0 | 1 | 1 | 0 | 0 | 0 |

Supplementary Table S5 (Excel) Information of taxonomy and abundance for KOs showing in figure 5.

Supplementary Fig. S1 Rarefaction curves of orthologous genes in metagenomic (A) and metaproteomic (B) datasets.


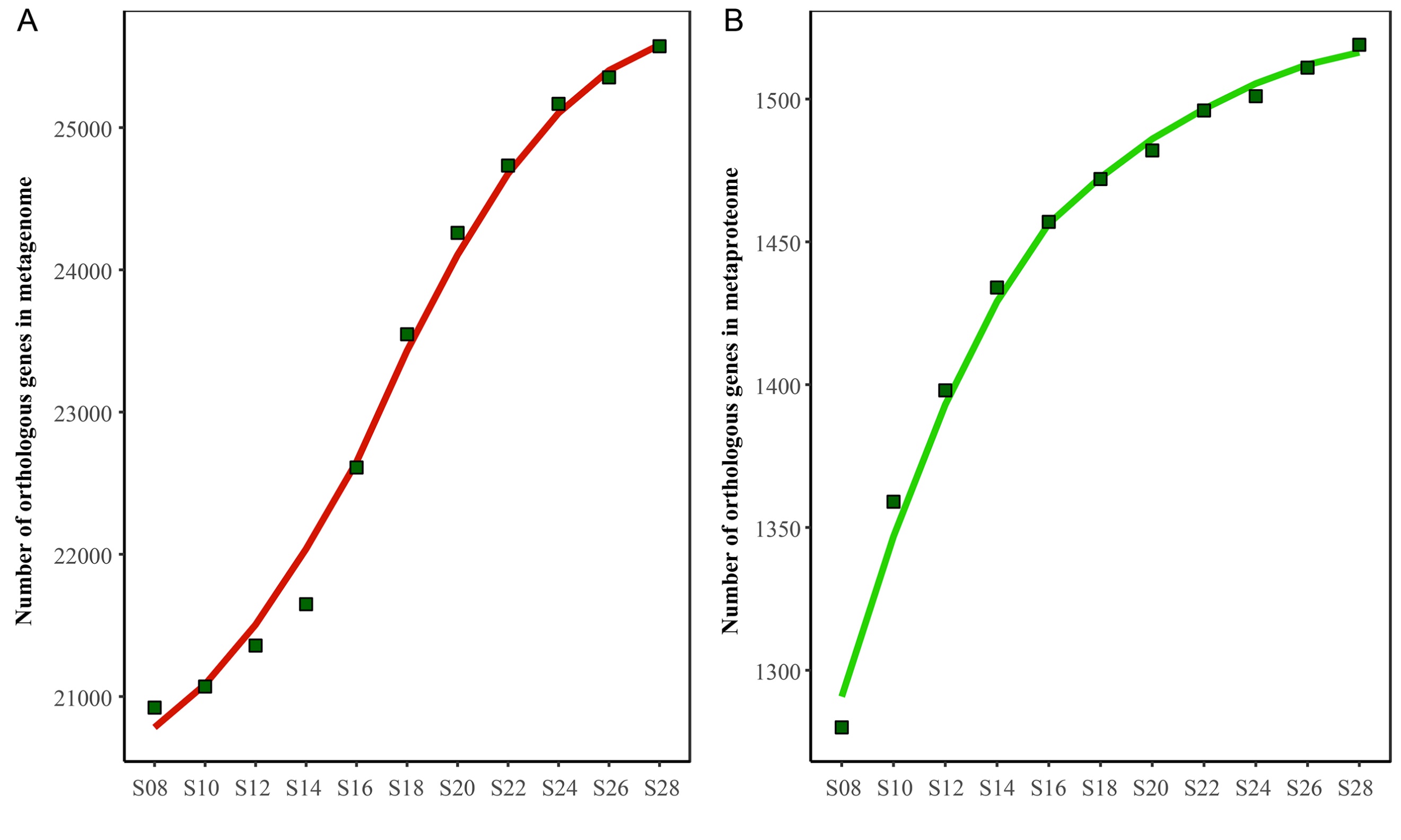


Supplementary Fig. S2 Percentages of core and non-core OGs in terms of numbers (A) and relative abundances (B) in metagenome and metaproteome.


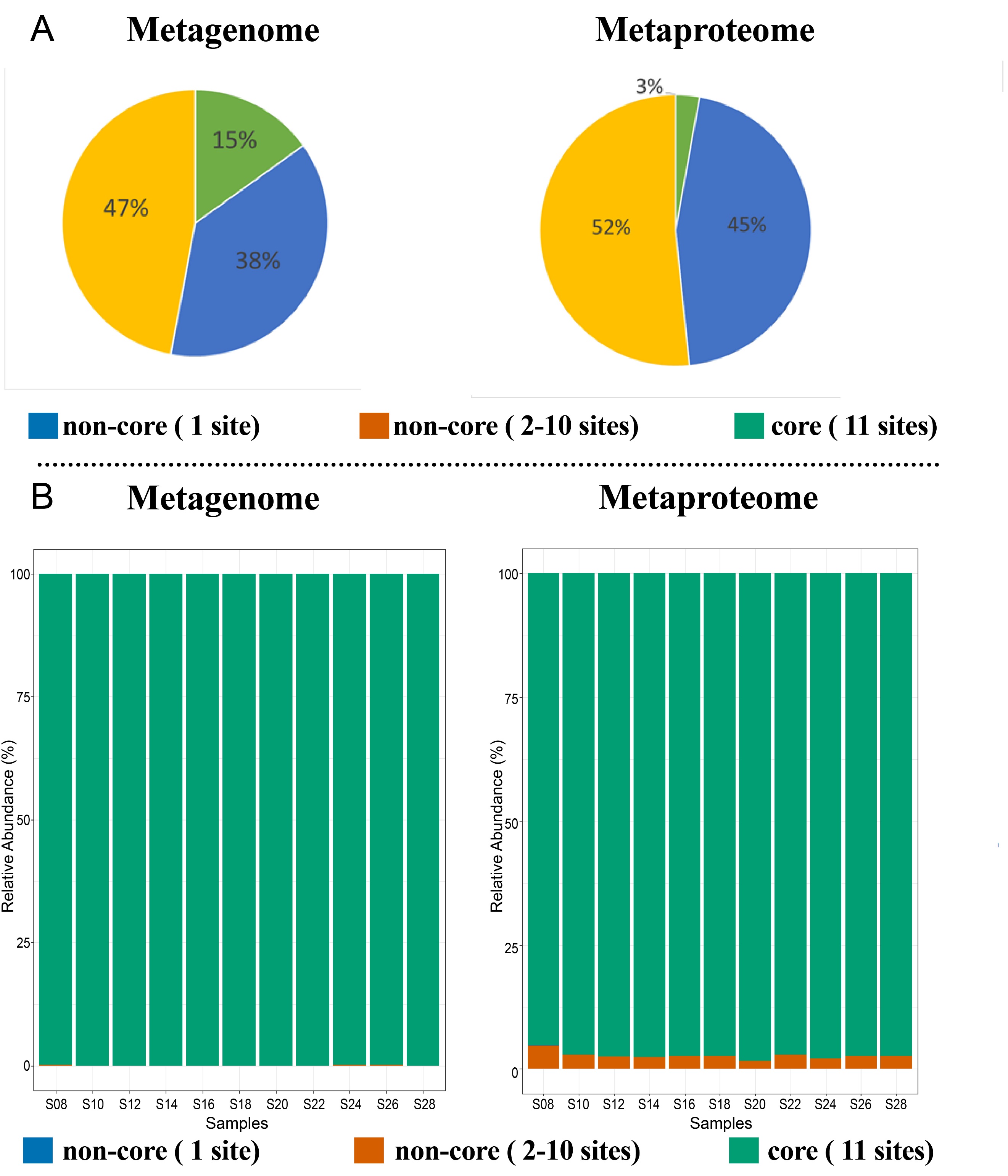


Supplementary Fig. S3 Spearman correlation analysis on metagenome (A & C) and metaproteome (B & D) showing genera groups significantly correlated to salinity (A & B) and temperature (C & D).


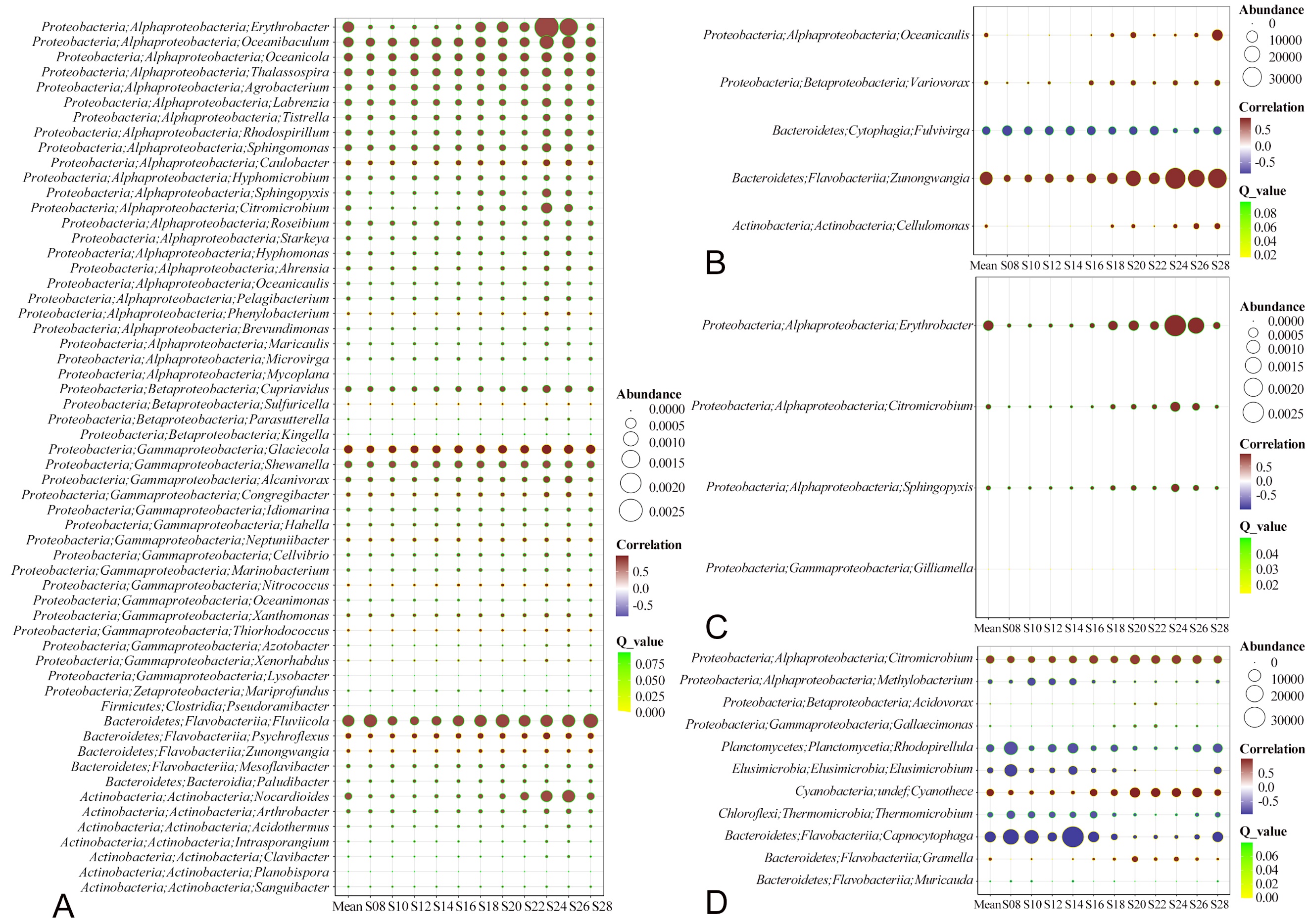

Supplement: Supplementary file 2 — Supplementary file2 (DOCX 2434 KB) [file 42995_2021_119_MOESM2_ESM.docx]
